# Pinhole-free 2D Ruddlesden–Popper perovskite layer with close packed large crystalline grains, suitable for optoelectronic applications

**DOI:** 10.1038/s41598-023-35546-1

**Published:** 2023-05-24

**Authors:** Parsa Darman, Amin Yaghoobi, Sara Darbari

**Affiliations:** Nano-Sensors and Detectors Lab., and Nano Plasmo-Photonic Research Group, Faculty of Electrical and Computer Engineering, Tarbiat Modares University, Tehran, Iran

**Keywords:** Optoelectronic devices and components, Two-dimensional materials

## Abstract

Here, we achieved pinhole-free 2D Ruddlesden–Popper Perovskite (RPP) BA_2_PbI_4_ layers with close packed crystalline grains with dimension of about 30 × 30 µm^2^, which have been demonstrated to be favorable for optoelectronic applications, such as fast response RPP-based metal/semiconductor/metal photodetectors. We explored affecting parameters in hot casting of BA_2_PbI_4_ layers, and proved that oxygen plasma treatment prior to hot casting plays a significant role to achieve high quality close packed polycrystalline RPP layers at lower hot cast temperatures. Moreover, we demonstrate that crystal growth of 2D BA_2_PbI_4_ can be dominantly controlled by the rate of solvent evaporation through substrate temperature or rotational speed, while molarity of the prepared RPP/DMF precursor is the dominant factor that determines the RPP layer thickness, and can affect the spectral response of the realized photodetector. Benefiting from the high light absorption and inherent chemical stability of 2D RPP layers, we achieved high responsivity and stability, and fast response photodetection from perovskite active layer. We achieved a fast photoresponse with rise and fall times of 189 µs and 300 µs, and the maximum responsivity of 119 mA/W and detectivity of 2.15 × 10^8^ Jones in response to illumination wavelength of 450 nm. The presented polycrystalline RPP-based photodetector benefits from a simple and low-cost fabrication process, suitable for large area production on glass substrate, a good stability and responsivity, and a promising fast photoresponse, even around that of exfoliated single crystal RPP-based counterparts. However, it is well known that exfoliation methods suffer from poor repeatability and scalability, which make them incompatible with mass production and large area applications.

## Introduction

Two dimensional Ruddlesden–Popper organo-metal halide Perovskite (RPP) layers are quantum well-structured materials, which have been applied first in solar cells in 2015, leading to an efficiency of 4%. Then, following research groups have pursued this field and enhanced the efficiency of RPP-based solar cells up to 21% in 2021^1–4^. Moreover, RPP layers benefit from a significantly enhanced stability in their optoelectronic operation, which entitles them as one of the solutions for the stability challenge in bulk perovskite layers^5^. Up to now, promising realizations of photodetectors, LEDs, and lasers have been reported based on RPP materials^6–10^, so that they seem attractive candidates for future optoelectronic applications^11^. In the Ruddlesden–Popper phase a spacer molecule (barrier) isolates certain number of perovskite layers (well) along the z axis, serving as a surrounding capsule that improves stability by preventing penetration of humidity and other species to the perovskite layers^12,13^. The general formula of organo-metal halide perovskite Ruddlesden–Popper phase is (R-NH3)_2_(A)_n−1_M_n_X_3n+1_, where R-NH_3_ is a hydrophobic large aliphatic or aromatic alkylammonium spacer cation. A is a monovalent cation sitting at the center of perovskite cubic structure and can be occupied with MA and FA. M stands for metals like Pb, Sn, and Ge, making ionic bond with halogens like I, Br, and Cl at the X site, and forming octahedrons with metal at the center and halogens at each corner that are responsible for high photo-absorption coefficient in RPP layers^13–15^. n is the number of octahedral layers between the spacer molecules, which determines the well width in the quasi-two dimensional structures of RPP layers. Multiple layers of metal halide octahedral can be formed with a cubic lattice structure with A atoms at the center and the aforementioned octahedrons at each corner of the cube. The barrier width between the layers in these quantum cascade structures is determined with the size of the spacer molecule and the width of alkyl ligand^13,16–18^.

Regarding the optoelectronic properties of RPPs, Ishihara et al. reported an extraordinary exciton binding energy of about few hundred meV for (C_n_H_2n+1_NH_3_)_2_PbI_4_, which was attributed to the quantum confinement, and dielectric confinement in these 2D layers^18–22^. Regarding the dielectric confinement in RPPs, smaller dielectric coefficient of barriers than the wells reduces the screening effect of barriers, leading to stronger electron–hole Columbic interactions all over the wells, and stronger exciton binding energy consequently^18,23^. Considering the attractive optoelectronic properties of RPPs, numerous research groups have reported different applications for them. In this line of research, Kondo et al.^24^ reported biexciton lasing in (C_12_)_2_PbI_4_. Scompus et al. reported a complete investigation of (BA)_2_(MA)_n−1_Pb_n_I_3n+1_ from n = 1 to ∞, and studied their performance in solar cells, while Dehghani et al. investigated nonlinear optics properties of RPP layers with n = 1,2^1,11,13^. Tsai et al. deposited RPP layers by hot casting, which improved power conversion efficiency (PCE) of RPP-based solar cells from 4 to 12%^5^. Besides, they proved that layer thickness plays a key role in PCE of solar cells with vertical configuration and vertical carriers transport, due to barriers effect on carrier recombination^5,25–27^. In optoelectronic devices with lateral configuration, carriers transport through corner sharing octaherdas, without experiencing the barrier layers, leading to a superior charge transport expectation. However, this expectation did not occur in practice, because of the carrier scattering at the crystal grain boundaries with high trap densities^6,28,29^. Zhou et al. reported a lateral photoconductor, based on spin coated (BA)_2_(MA)_n−1_Pb_n_I_3n+1_ with n = 1, 2, and 3, using Au contacts^30^, resulting in responsivities of 3, 7.31, and 12.78 mA/W and rise (fall) times of 28.4 (27.5) ms, 8.4 (7.5) ms, and 10 (7.5) ms, for n = 1, 2, and 3, respectively. Loi et al. fabricated lateral phototransistors, based on hot cast (PEA)_2_(MA)_n−1_Pb_n_I_3n+1_ with vertical heterostructures of RPP layers, wherein n gradually changes from 1 at the bottom to ∞ at the top of the film. They achieved outstandingly high responsivity of 149 A/W, and rise (fall) time of 69 (103) ms^31^. Hwang et al. utilized hot casting method to fabricate RPP-based photodetectors, using (C_4_H_9_NH_3_)_2_(CH_3_NH_3_)Pb_2_I_7_, and achieved responsivity of 8.4 A/W, and detectivity of 1.2 × 10^12^ Jones^32^. To date, all reported poly crystalline RPP layers suffer from low crystalline quality and pinholes or defects, which have led to photoresponse times of ms-scale, well below the reports based on exfoliated single crystal RPPs. However, it is well known that exfoliation methods suffer from poor repeatability and scalability, which make them incompatible with mass production and large area applications.

Here, we report a pinhole-free 2D Ruddlesden–Popper Perovskite (RPP) BA_2_PbI_4_ layer with close packed crystalline grains with dimension of about 30 × 30 µm^2^, suitable for realizing efficient optoelectronic applications, such as fast response RPP-based metal/semiconductor/metal photodetectors, with high responsivity and repeatability. We investigate the effect of domiant parameters on the quality of polycrystalline RPP layers, including grain size, pinholes and morphology, optical properties, and photodetection performance. We present the effects of dominant parameters in hot casting of BA_2_PbI_4_ layers, including oxygen plasma pre-treatment, substrate temperature, spin coating rotational speed, and molarity of the RPP/DMF precursor on the quality of close packed polycrystalline RPP layers at lower hot cast temperatures, the crystal growth and grains sizes, and the RPP layer thickness, respectively. We optimize the hot casting parameters to achieve pinhole-free RPP layer with close packed and μm-scale grains, which leads to effective transport of photogenerated carriers to metal electrodes, resulting in high responsivity and fast response, in addition to stable photoresponse owing to the inherent chemical stability of 2D RPP layers. Our achieved response times from photodetectors based on poly crystal RPP layers, are significantly higher than the previously reported hot cast poly crystalline RPP layers, while comparable with that of exfoliated single crystal RPP-based counterparts, which suffer from poor repeatability and scalability^30,31,33–35^. In the presented RPP layer synthesis method, process investiagtion and the resulting photodetection results, we propose a simple and low-cost fabrication process of efficient and well-controlled 2D RPP layers on glass substrate, which overcome the challenges of repeatability, large area and mass production in exfoliated 2D single crystal RPP layers, while achieving fast response, high responsivity, high repeatability and acceptable stability.

## Results and discussion

**Figure 1:**
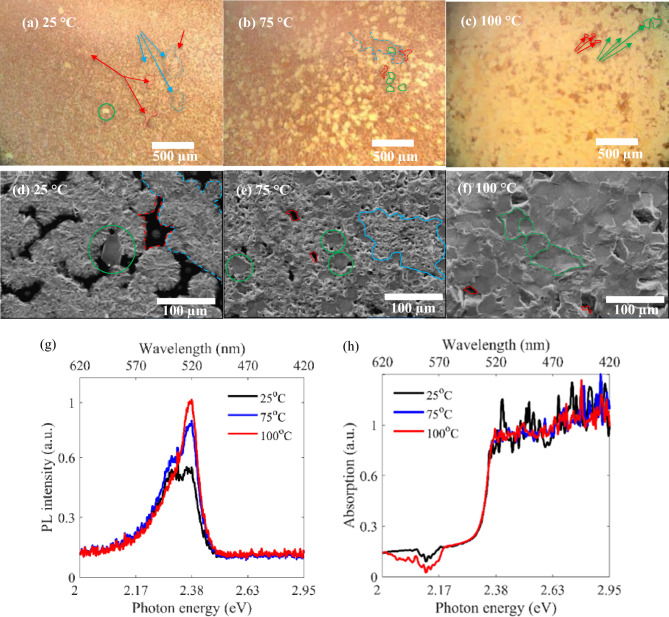
(**a**–**c**) Optical microscopy images, (**d**–**f**) SEM images, (**g**, **h**) PL and absorption spectra of RPP layers prepared at hot casting temperatures of 100 °C, 70 °C, and 25 °C.

To study the effect of perovskite hot casting conditions on the morphology and optical properties of the layers, first we investigate the hot casting temperature. For this purpose, we heated two substrates at 100 °C and 70 °C for 10 min before spin coating, while the third substrate is kept at room temperature (25 °C). Now, we spin coated a 1.8 molar solution of RPP/DMF with rotational speed of 3000 rpm for 40 s on the substrates. Figure 1a–c show the top view optical images of the layers prepared at room temperature, 70 °C, and 100 °C, while Fig. 1d–f illustrate the corresponding SEM images, respectively. Green circles enclose the isolated crystalline grains in Fig. 1a,d,e, while the increased number of crystalline grains are highlighted by green boundaries in Fig. 1b,c,f, which are forming close packed crystalline grains that cover the surface dominantly. Moreover, red and blue boundaries and arrows correspond to the pinholes and amourphous regions between the crystalline grains, respectively. It can be observed that for lower temperature (Fig. 1a), there are few crystal grains (green boundaries) with bright optical transmission vision in the achieved RPP layer, while the amorphous regions (blue boundaries) with randomly scattered transmission light are evident in optical images, due to existence of considerable surface roughness. Furthermore, it is observable in this figure that increasing the heating temperature leads to larger crystalline grains with lower overall surface roughness, so that the neighboring grains in the layer pack together and cover mostly the whole sample area. Figure 1f reveals a mean crystal grain size of about 30 × 30 µm^2^ for hot casting at 100 °C. This improved crystal growth is also observable in the relating PL spectra (Fig. 1g), illustrating higher peak intensity at 517 nm for RPP layers prepared at higher temperatures. This main PL peak (at 517 nm) is attributed to emissive free excitons in the prepared RPP layers^1,36^. However, we proved that room temperature spin coating has led to layers with rough surfaces in Fig. 1a,b,d,e, which correspond to a secondary radiative recombination PL peak at 538 nm in Fig. 1g. In this regard, some researchers suggested a localized exciton nature for a similar peak in their PL analysis of RPP layers^17,37–39^, for instance, Chong et al. attributed this peak to the surface bound excitons in RPP layers^38^. Considering these reports and the discussed roughness in low temperature samples, we attribute our secondary PL peak (538 nm) to the surface bound excitons. It is obviously observed in Fig. 1g that the secondary peak is disappeared in PL spectrum of higher temperature sample, because of the improved morphology and passivated surface defects (shown in Fig. 1c,f)^40,41^. The recorded absorption spectra of the investigated samples (Fig. 1h) show almost a consistent behavior, revealing an absorption edge at 517 nm for all the prepared polycrystalline RPP layers.

**Figure 2:**
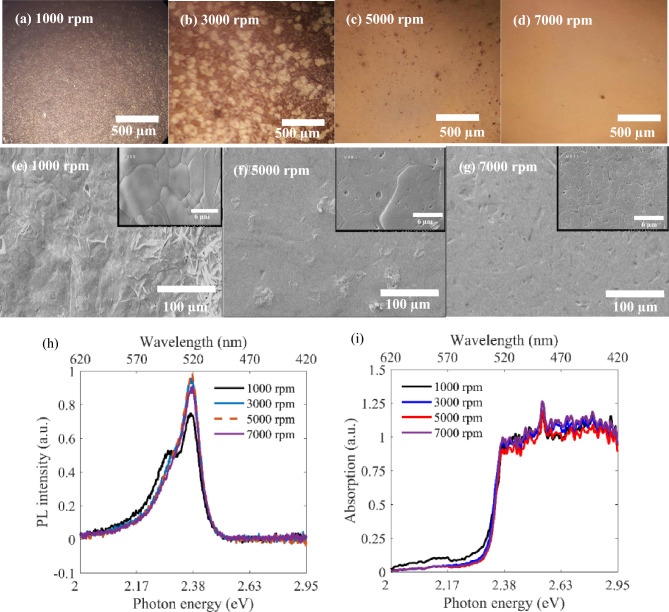
(**a**–**d**) Optical microscopy images of RPP layers hot cast at rotational speed of 1000 rpm, 3000 rpm, 5000 rpm, and 7000 rpm. (**e**–**g**) SEM images relating to samples, prepared at 1000 rpm, 5000 rpm, and 7000 rpm. The insets show a magnified view of the crystalline gains in the layers. (**h**, **i**) PL and Absorption spectra of RPP layers corresponding to 1000 rpm, 3000 rpm, 5000 rpm, and 7000 rpm.

Then, we studied the effect of rotational speed in spin coating of a 1.8 molar RPP/DMF solution, at a fixed hot cast temperature of 100 °C. Figure 2a–d illustrate the optical microscopy images of the prepared (BA)_2_PbI_4_ layers, spin coated with 1000 rpm, 3000 rpm, 5000 rpm, and 7000 rpm, respectively. Considering that bright regions and dark regions correspond to crystalline grains and amourphous regions, we can assume the bright to dark surface area ratio as crystallization ratio, which is evidently increased from part (a) to part (d) of Fig. 2. These figures indicate that layer morphology, uniformity, and crystal growth have been improved significantly for higher rotational speeds. It is observable that increasing the rotational speed from 1000 to 5000 rpm, leads to larger and more close packed crystal grains, covering the whole surface area. However, Fig. 2d indicates a macroscopically uniform layer for 7000 rpm, wherein grain boundaries are not optically evident. Figure 2e–g display the SEM images relating to samples of 1000 rpm, 5000 rpm, and 7000 rpm, confirming larger close packed grains for 5000 rpm rather than 1000 rpm, and proving that increasing the rotational speed up to 7000 rpm leads to smaller micro-crystals, in contrary to the trend for rotational speeds lower than about 5000 rpm. The insets in Fig. 2e–g clarify the crystal grain conditions for each rotational speed in hot casting. Figure 2h,i show the PL and absorption spectra of the layers prepared with different rotational speeds, respectively. It can be observed that there is no distinguishable difference between the absorption behaviors, all showing the same absorption edge at 517 nm, which is in agreement with Fig. 1h. PL spectra in Fig. 2h exhibit that rotational speeds higher than 3000 rpm lead to nearly similar main peaks at 517 nm, while for lower rotational speeds a secondary radiative recombination peak is observable at around 538 nm, which is again attributed to higher surface recombination due to surface roughness and defects, similar to Fig. 1g. According to the aforementioned discussions, this secondary trap-assisted peak is demolished for higher rotational speeds, due to better crystal growth. Therefore, we found 5000 rpm as the optimum rotational speed in our RPP hot casting, since the layer was entirely consisting of close packed crystal grains with dimensions of about 30 × 30 µm^2^, and the relating PL peak intensity is maximized simultaneously, indicating less probable Shockley–Read–Hall recombination due to less surface roughness and defects. From the photodetection aspect of view, as our main objective in this work, better grain interfaces in the prepared layer improves carrier transport, and photodetection performance consequently^32,42^.

**Figure 3:**
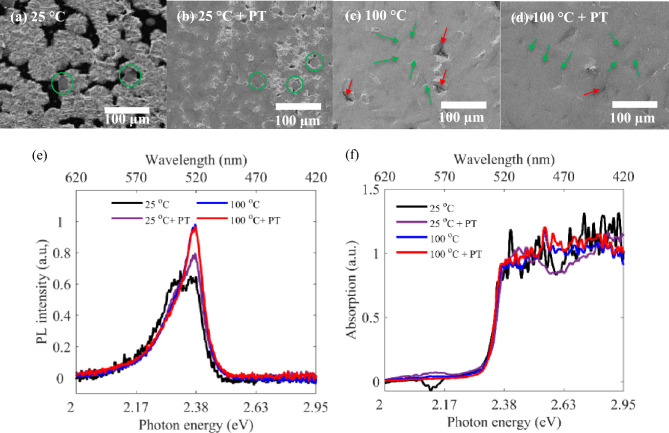
(**a**–**d**) SEM images relating to samples (**a**) spin coated at 25 °C, (**b**) exposed to PT before spin coating at 25 °C, (**c**) hot cast at 100 °C, and (**d**) exposed to PT before hot casting at 100 °C. (**e**) Absorption, and (**f**) PL spectra of the investigated RPP layers.

As the next stage, we have studied the effect of oxygen plasma treatment (PT) of substrate prior to hot casting. Figure 3a–d display SEM images relating to samples (a) spin coated at 25 °C, (b) plasma treated before spin coating at 25 °C, (c) hot cast at 100 °C and (d) plasma treated before hot casting at 100 °C. Green circles in Fig. 3a,b enclose the isolated crystalline grains, and green arrows in Fig. 3c,d show the trace of grain boundaries between the close packed crystalline grains, while red arrows correspond to the trace of pinholes. It can be observed that surface morphology and crystallization of the layers has been improved after PT and the achieved layers have converted to a pinhole-free poly crystalline RPP layer with minimized roughness and defects at the grain boundaries, at both temperatures of 25 °C and 100 °C. However this enhancement is more significant at lower temperatures. In other words, utilizing an initial oxygen PT can compensate lack of enough thermal energy in hot casting, allowing high quality polycrystalline RPP layers at lower temperatures. The observed enhanced surface morphology by applying an initial PT, is in accordance with the measured PL spectra in Fig. 3e, wherein the secondary PL peak at 538 nm, relating to surface traps has been suppressed and the main peak at 517 nm has been boosted for samples with PT. On the other hand, the corresponding absorption spectra in Fig. 3f show no distinguishable difference between the investigated samples, all confirming an absorption edge at 517 nm, the same as in Figs. 1h and 2i. As oxygen plasma treatment makes the substrate more hydrophilic, the improved crystal growth can be explained by the reduced contact angle of RPP/DMF precursor solution, resulting in an increased solvent evaporation rate^43^. Spreading enhancement of the precursor solution on the substrate, being exposed to the air environment, leads to solvent evaporation and supersaturation, crystal nucleation and enhanced crystal growth with aligned orientations in the layer^44^. Our presented characterizations prove that the substrate temperature, rotational speed and plasma treatment in hot casting affect the crystallization and grain size of the resulted RPP layers significantly, the observation which is attributed to the affected solvent evaporation rate and consequent supersaturation degree. Our observations are in agreement with previous reports, wherein higher degree of supersaturation at intermediate saturation range, has led to higher nucleation and crystal growth rate^32,44,45^; however, further increasing the crystallization rate has led to RPPs with smaller crystal grains^44^.

**Figure 4:**
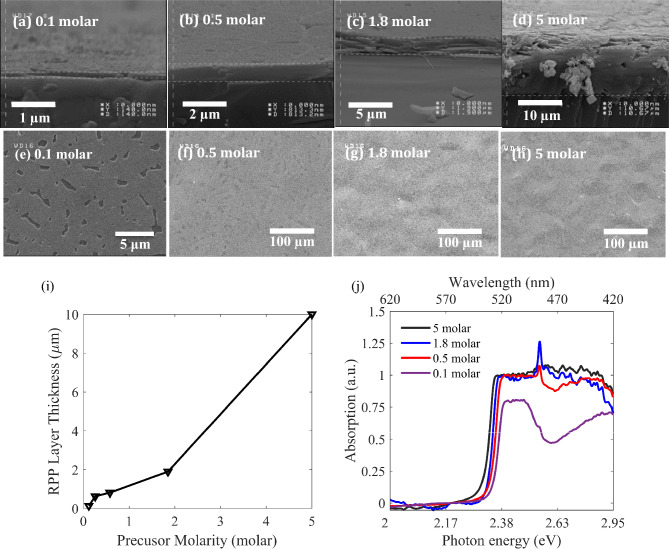
(**a**–**d**) Cross section view, and (**e**–**h**) top view SEM images of RPP layers, hot cast by RPP/DMF solutions of 0.1 molar, 0.5 molar, 1.8 molar, and 5 molar. (**i**) Variation of layer thickness versus increasing the molarity of RPP/DMF solution. (**j**) Absorption spectra of RPP layers, hot cast by RPP/DMF precursors of 5 molar, 1.8 molar, 0.5 molar, and 0.1 molar.

**Figure 5:**
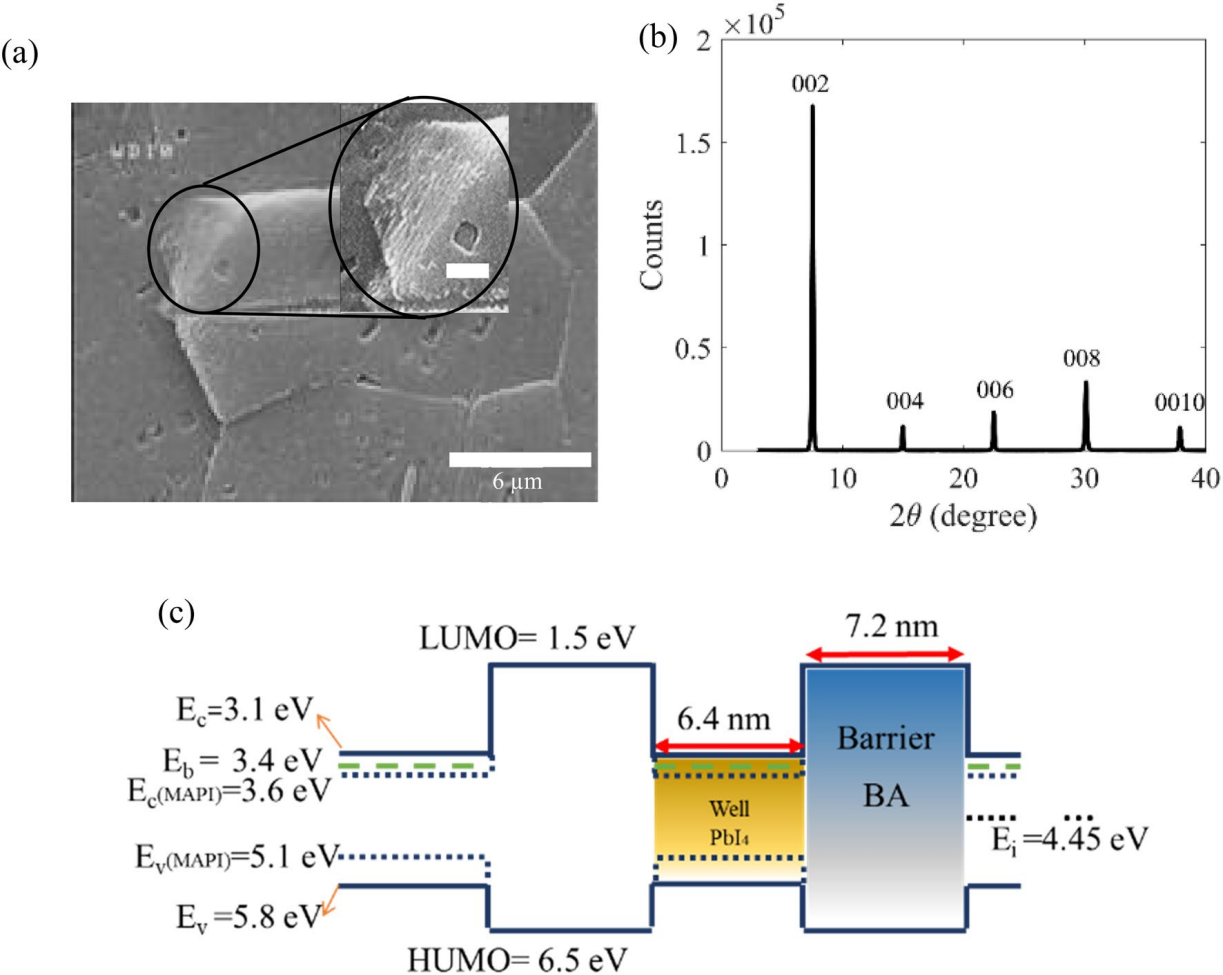
(**a**) SEM image of a crystalline grain in the realized RPP layer, revealing multiple stacks of perovskite layers parallel to the substrate surface. Scale bar in the inset is 600 nm. (**b**) XRD analysis of the realized polycrystalline RPP layer. (**c**) The proposed band diagram for the achieved RPP layer with n = 1 along the vertical direction, consisting of periodic quantum wells of PbI_4_ and barriers of BA.

To investigate the effect of RPP/DMF solution molarity on the achieved hot cast layers, we use different molarities for spin coating at the fixed rotational speed of 5000 rpm and hot cast temperature of 100 °C. We present the results of four different cases in Fig. 4, wherein parts (a–d) show cross section view and parts (e–h) indicate top view SEM images, corresponding to RPP/DMF solution of 0.1 molar, 0.5 molar, 1.8 molar, and 5 molar, respectively. It is observed that RPP layer thickness is evidently increased for higher RPP/DMF solution molarities, as demonstrated by layer cross sections in Fig. 4a–d. Figure 4i indicates the variations of layer thickness versus different molarities of RPP/DMF solution, wherein thicknesses of 10 μm, 1.9 μm, 0.81 μm, 0.62 μm and 0.14 μm correspond to precuresur molarities of 5 molar, 1.85 molar, 0.58 molar, 0.26 molar and 0.12 molar, respectively. In contrast, we showed that hot cast temperature and rotational speed in the aforementioned investigated ranges do not change the layer thickness and the absorption behavior significantly, as shown in Figs. 1 and 2. Parts (f–h) of Fig. 4 indicate that morphology of the hot cast layers is not sensitive to precursor molarity, specially for molarities higher than about 0.5 molar. However, decreasing molarities lower than 0.5 molar leads to increasing pinholes (Fig. 4e), and conversion of close packed large crystal grains to rather isolated small crystal grains (as shown in Fig. S1). Figure S1 confirms that further decreasing the solution molarity lower than 0.1 molar leads to formation of isolated perovskite crystal islands with optical properties of which are presented in this figure. The presented data in Figure S1 are consistent with previous reports at similarl synthesis conditions^46^. The isolated crystal islands, achieved from molarities lower than 0.1 molar are not suitable for the active layer in photodetection applications. Hence, we concentrated on precursor molarities higher than about 0.1 molar in the present work. Figure 4j displays the absorption spectra for different solution molarities, proving a blue shift in the absorption edge from 534 to 520 nm versus decreasing the precursor molarity from 5 molar to 0.5 molar. Further decreasing the precursor molarity down to 0.1 molar, leads to further blue shift of the absorption edge to 517 nm, and emergence of a relative absorption dip around 470 nm. This observed absorption dip will be discussed based on the band structure of RPP layer (Fig. 5). Considering the absorption spectrum of 0.1 molar (violet curve) in Fig. 4j, we can calculate the absorption coefficients of 1.15 × 10^5^ cm^−1^, 4.75 × 104 cm^−1^, and 8.87 × 10^4^ cm^−1^ at wavelengths of 515 nm, 475 nm, and 420 nm, respectively, which are approximately consistent with previous reports^5^.

Figure 5a, b show the magnified view SEM image and XRD analysis of the RPP layer prepared from a solution of 1.8 molar, hot cast at 100 °C, and spin coated with 5000 rpm. The inset in Fig. 5a reveals the stack of perovskite monolayers in the layer, which have grown parallel to the substrate. According to the achieved XRD spectrum in Fig. 5b, every peak corresponds to (0 0 2l) planes, wherein l is integer^13^, and the spacing between the crystal planes is measured *d* = 13.6 nm (Table S1). Regarding these, we propose the periodic band diagram across the layer thickness of our prepared RPP layers (n = 1) in Fig. 5c, wherein BA layers play as insulator barriers between the PbI_6_ monolayers. The proposed periodic potential well is consistent with the relating previous studies^47^. The spacing between the crystal planes or the thickness of one stack in our RPP layer equals the summation of thicknesses of a single PbI_6_ monolayer and a single barrier (BA) layer. Since there is one layer of PbI_6_ octahedra in our prepared RPP layers (n = 1), the well width equals the sum of two Pb-I ionic bonds (6.4 nm), and the barrier length is achieved 7.2 nm = *d*-6.4 nm^13,18^. Moreover, the lowest unoccupied and highest occupied states of BA (barrier) correspond to 1.5 eV and 6.5 eV, respectively^47^. On the other hand, the electron affinity and ionization energy of MAPbI_3_, as the bulk limit case (n → ∞) of RPP layers (BA)_2_(MA)_n−1_Pb_n_I_3n+1_, correspond to 3.6 eV and 5.2 eV, respectively. Regarding these bulk energy levels, Silver et al. have used Kronig-Penney method to calculate the ground states of conduction and valence bands in the RPP layer with n = 1 equal to 3.1 eV and 5.8 eV. Moreover, they calculated the exciton binding energy (E_B_) and exciton energy level as 300 meV and 3.4 eV, respectively^47^. Here, we have used the previously reported energy levels in band diagram of Fig. 5c. Regarding the proposed band diagram, we explain the previously observed absorption spectrum of 0.1 molar sample (Fig. 4j), by two absorption mechanisms: (i) band-to-band transition in the quantum well from 5.8 to 3.1 eV, responsible for the absorption of photon energies (wavelengths) higher (lower) than about 2.7 eV (460 nm), and (ii) free exciton absorption, leading to a peak at energies (wavelengths) around 2.7 eV − E_B_ = 2.4 eV (≈ 517 nm). The latter absorption peak is consistent with our previously described free exciton PL peaks (in Figs. 1g and 2h). It should be noted that further reducing the solution molarity (below 0.1 molar) leads to sharper free exciton peaks at the same energy, coming dominant in the absorption spectra (Fig. S1l). On the other hand, BA is the shortest possible alkyl ammonium group as the barrier in the Ruddlesden–Popper phase (with a chain of 4 carbon atoms)^21^, leading to a strong coupling between the quantum wells in Fig. 5c. This coupling leads to formation of a super lattice-like behavior by increasing the number of quantum wells in the layer, which corresponds to layers prepared from higher precursor solution molarities. In other words, tunneling resonance in thicker RPP layers with n = 1, results in band broadening and merging of both the band-to-band and free exciton absorption peaks, so that the absorption dip of the violent spectrum (Fig. 4j) at about 2.63 eV (470 nm) disappears gradually by increasing the precursor molarity and layer thickness in the red, blue, and black spectra^41,48–53^.

**Figure 6:**
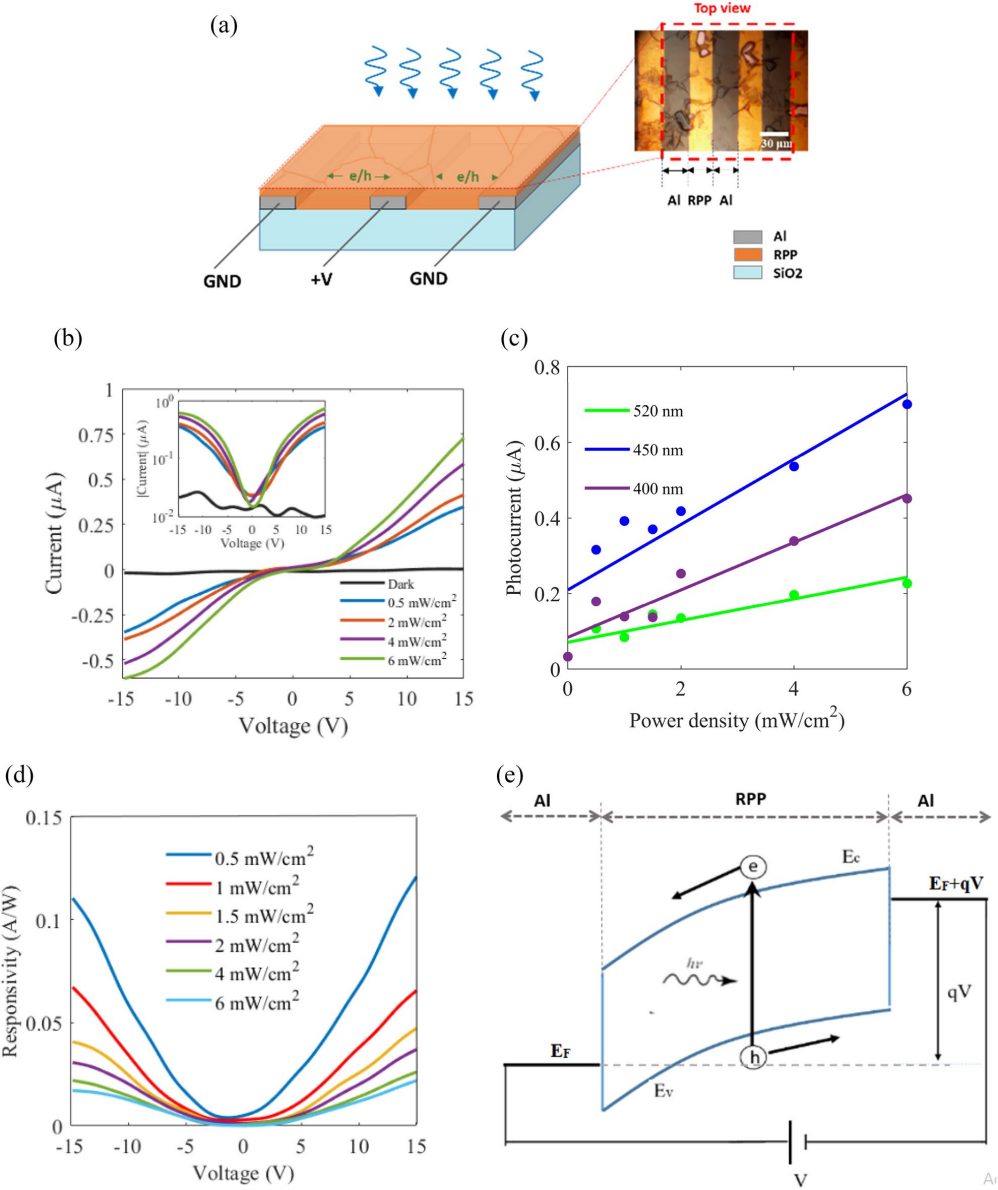
(**a**) Schematic illustration of the fabricated lateral MSM photodetector based on the achieved optimized poly crystalline RPP layer, wherein grain boundaries are observable. The magnified view shows the optical microscopy image of the RPP layer on the metal interdigital electrodes. (**b**) The current–voltage characteristics of the fabricated Al/RPP/Al photodetector in response to different illumination power densities at wavelength of 450 nm. The inset shows the current in logarithmic scale. (**c**) The measured photocurrent versus different power densities of green (520 nm), blue (450 nm), and UV (400 nm) illuminations, at a fixed bias voltage of 15 V. (**d**) The calculated responsivities versus different bias voltages, for different illumination powers at 450 nm. (**e**) Band diagram of the fabricated Al/RPP/Al photodetector along the lateral direction, when a bias voltage is applied between the electrodes.

Considering our investigated absorption spectra, the red spectrum in Fig. 4j, corresponds to the minimum thickness (800 nm) of RPP layer that absorbs the whole wavelength spectrum below 517 nm. In other words, solution molarity of 0.5 molar is the least molarity that does not show significant dip in the absorption band (λ < 517 nm). Thus, we expect that RPP layers thinner than 800 nm lead to lower photodetection responsivity due to their lower absorption levels. On the other hand, we expect that thicker layers lead to weaker carrier transport through the thick stack of alternating BA/PbI_6_ layers and lower responsivity, consequently. Therefore, we expect the best photodetection behavior from hot casting of about 0.5 molar RPP/DMF solution, at 100 °C with rotational speed of 5000 rpm in metal/RPP/metal structure. Figure 6a displays the fabricated photodetector schematically, wherein the bottom PbI_6_ monolayers of octahedra in the stack can be laterally connected to metal electrodes, being partially isolated from each other by BA barriers. It demonstrated that the mean crystalline grain dimensions are comparable with the gap between metal electrodes, which can lead to a high responsivity and fast response. Photogenerated carriers around the top of the RPP layer may be collected through both vertical transport by tunneling through the BA barriers, and lateral 2D transport through the PbI_6_ monolayers. The magnified view shows the the top view optical image of the active region in the fabricated metal/RPP/metal photodetector, wherein the bright lines correspond to the interdigital electrodes, and the boundaries of the high quality micro-crystal grains in the RPP film are observable. Considering the observable mean grain size of about 30 μm in the realized RPP layer, we have fabricated interdigital electrodes with nearly the same electrode gap in order to benefit from the 2D lateral transport in PbI_6_ monolayers with minimal carrier scattering in the grain boundaries and defects.

Then, we utilized and studied two different metal contacts, Au and Al, in our metal/RPP/metal photodetector. Figure S2 shows the I–V characteristics of the fabricated Au/RPP/Au photodetector at the dark and green-illumination conditions. The measured linear I–V characteristics reveal an ohmic contact between Au and RPP layer that is in agreement with the relating previous reports^7,30–33,54–56^. The resulting resistive behavior in the ohmic contacts of the realized metal/RPP/metal structure in addition to the resistance of the RPP layer leads to a relatively lower equivalent resistance for the investigated photodetector, and a higher dark current consequently. The measured high dark current diminishes the detectivity for the realized Au/RPP/Au photodetector. However, replacing the ohmic contacts with Schottky contacts in metal/RPP/metal structure results in two back-to-back Schottky diodes in series with the resitance of RPP layer, which in turn leads to a nonlinear current–voltage characteristics and a relatively lower dark current. Moreover, Schottky contacts lead to formation of depletion regions in RPP layer and drifting of photogenerated carriers, which in turn decreases the transit time of carriers toward electrodes and suppresses carriers recombinations. Thus, realizing metal/RPP/metal configuration with Schottky contacts would lead to higher photocurrents with respect to the dark current. Regarding this, replacing Au by Al, Fig. 6b displays the achieved I–V characteristics for dark condition, and illumination wavelength of 450 nm with different powers from 0.5 to 6 mW/cm^2^. It is observable that the dark current is significantly lower than the photocurrents, and the optoelectronic characteristics reveal a nonlinear and symmetric I–V behavior, so that the photocurrents rise drastically at about 2.5 V. The observed nonlinearity in I–V characteristics is attributed to the Schottky behavior of Al/RPP contacts. The inset in Fig. 6b displays the absolute photocurrent values in logarithmic scale, versus the applied voltage. In Fig. 6c, the measured photocurrent (*I*_*ph*_) is plotted versus different illumination power densities (*P*_*in*_) for three different wavelengths, at the same bias voltage of 15 V. This plot indicates the highest photocurrents for blue illuminations, while the most linear *I*_*ph*_*–P*_*in*_ characteristic corresponds to green illumination in the fabricated device. The photodetection responsivity is calculated by $\frac{{I}_{ph}-{I}_{dark}}{{P}_{in}\times A}$, wherein *A* is the active area of the photodetector (*A *≈ 600 µm × 1800 µm) and *I*_*dark*_ is the dark current^29,57^. The achieved blue responsivity is plotted versus the applied voltage for various illumination power densities in Fig. 6d, confirming approximately a symmetric behavior for the positive and negative bias voltages, which is in agreement with the presented symmetric I–V characteristic, and is attributed to the symmetric structure and band diagram of the realized Al/RPP/Al photodetector. Moreover, Fig. 6d reveals that larger absolute voltages lead to higher responsivities, because of the increased reverse biasing of one of the back-to-back Schottky diodes in each applied voltage polarity. Figure 6e exhibits the proposed lateral band diagram and photodetection mechanism for the fabricated metal/semiconductor/metal (MSM) photodetector. E_C_ and E_V_ in this band diagram correspond to the conduction band and valence band of PbI_4_ well in Fig. 5c, E_F_ stands for Fermi energy level of metal contacts, q and V correspond to the electron charge and the applied voltage between the metal contacts. Figure 6e indicates that the photogenerated electron/hole in RPP layer are separated and drifted toward the positive and negative electrodes, respectively. It can be evidently deduced that utilizing the prepapred high quality RPP layers with large and close packed grains is crucial to achieve efficient carrier transport toward the contacts with minimal transit time and trapping chance, and a consequent fast response and highly responsive photodetection behavior^58–64^.

To prove our expectation about the best photodetection behavior from the optimized precursor molarity of 0.5 molar, we present the photoresponse characteristics of a similar Al/RPP/Al configuration, but using precursor molarity of 1.5 molar in Figure S3. Moreover, Fig. S4 display the optoelectronic characteristics of RPP-based photodetector with precursor molarity of 0.5 molar. It is observable that the achieved responsivity for molarity higher than 0.5 molar is well below the optimum molarity, which is in agreement with our previous assumption of weaker carrier transport through thicker stack of alternating BA/PbI_6_ layers.

**Figure 7:**
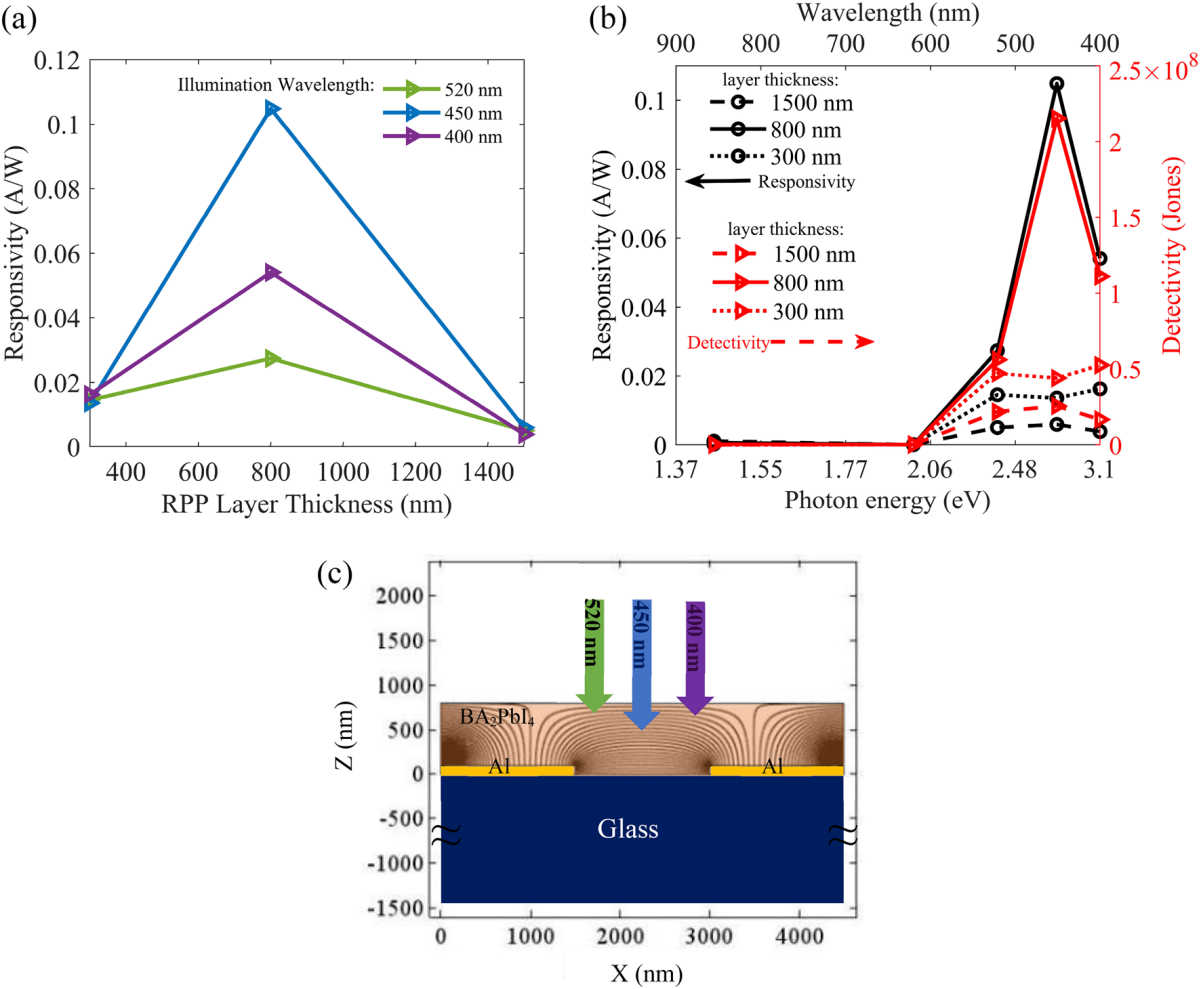
(**a**) Responsivities of the realized Al/RPP/Al photodetector to incident wavelengths of 400 nm, 450 nm, and 520 nm, versus different thicknesses of RPP layers, at a fixed power density of 0.5 mW/cm^2^ and bias voltage of 15 V. (**b**) Responsivity (black curve) and detectivity (red curve) variations at different incident wavelengths, for RPP layer thicknesses of 300 nm (dotted curve), 800 nm (solid curve), and 1500 nm (dashed-curve) at a fixed illumination power density of 0.5 mW/cm^2^. (**c**) Electric field distribution in the investigated Al/RPP/Al photodetector with bottom electrodes.

Then, we utilize different solution molarities to prepare different RPP layer thicknesses, and investigate the corresponding Al/RPP/Al photodetection responsivities. Figure 7a displays the resulting responsivity versus RPP layer thickness for incident wavelength of 450 nm at power density of 0.5 mW/cm^2^. It is observable that the maximum responsivity is achieved for the RPP layer thickness of about 800 nm, which is consistent with our previous discussions about the device design parameters including RPP layer thickness (see Figs. 4j and 5a). The maximum responsivity is measured 119 mA/W for the incident wavelength of 450 nm, and at power density of 0.5 mW/cm^2^, while decreases to 20.62 mA/W for power density of 6 mW/cm^2^, due to the carrier-carrier scattering and thermal effects^31,33,34,65–68^. It is notable that responsivity to wavelength of 450 nm for the layer thickness of 800 nm is much higher than the measured responsivity for thickness of 300 nm, for all incident powers. However, responsivities to 400 nm and 520 nm are nearly similar in the whole incident power density range. Black curves in Fig. 7b show responsivities versus illumination wavelengths (and photon energies), at the same incident power density of 0.5 mW/cm^2^. This plot reveals a nearly wavelength independent responsivity for the wavelength range of 400–520 nm, for the RPP layer thickness of 300 nm (black-dotted curve) and 1500 nm (black-dashed curve), while confirms the maximum photoresponsivity to 450 nm for the RPP layer thickness of 800 nm (black-solid curve). Hence, RPP layer thickness of 300 nm (or 1500 nm), corresponding to precursor of 0.17 molar (or 1.5 molar), can be used to realize a broadband Al/RPP/Al photodetector with a nearly uniform responsivity in the 400–520 nm wavelength range. The detectivity is also defined as $\mathrm{D}=\mathrm{R}/\sqrt{2{\mathrm{qI}}_{\mathrm{dark}}/\mathrm{A}}$, wherein R is the responsivity, *q* is the electron charge, and *A* is the active area^57^. The calculated detectivities reveal similar behaviors to the responsivities, as shown by red curves in Fig. 7b. Detectivity is maximum (2.15 × 10^8^ Jones) for incident wavelength of 450 nm at layer thickness of 800 nm and power density of 0.5 mW/cm^2^. Similarly, detectivity proves an approximate wavelength independent behavior for 300 nm (and 1500 nm) layer thickness. To explain the observed responsivity behavior in Fig. 7b, we should consider the photon penetration depth at each wavelength, and the applied electric field distribution across the RPP layer in the device. It is well known that the penetration depth in a material is wavelength dependent and is defined by ${\delta }_{p}=\frac{1}{\alpha }$, wherein α is the absorption coefficient. Utilizing absorption data in Fig. 4j, we had calculated absorption coefficients of the RPP layer at wavelengths of 400 nm, 450 nm, and 520 nm, achieving the corresponding absorption lengths of 113 nm, 153 nm, and 87 nm, respectively. To evaluate the optoelectronic behavior of the realized photodetector, we have numerically solved Poisson equations to achieve the electric field distribution in a unit cell, consisting of an electrode pair in our MSM photodetector (Fig. 7c), while assuming periodic boundary conditions. The effective dielectric constant of our RPP layer ((BA)_2_PbI_4_) can be estimated by $\upvarepsilon =\frac{{\upvarepsilon }_{\mathrm{b}}{\mathrm{L}}_{\mathrm{b}}+{\upvarepsilon }_{\mathrm{w}}{\mathrm{L}}_{\mathrm{w}}}{{\mathrm{L}}_{\mathrm{b}}+{\mathrm{L}}_{\mathrm{w}}}$=$3.98$, wherein ${\varepsilon }_{b}$=2.1 is the dielectric constant of barrier (BA), ${L}_{b}$=7.2 nm is the barrier width, ${\varepsilon }_{w}$=6.1 is the dielectric constant of the well (PbI_6_), and ${L}_{w}$=6.4 nm is the well width^18,23^. Considering the utilized bottom electrode configuration in our photodetector (Fig. 7c), for RPP layers thicker than around 800 nm, the electric field is evidently weaker (stronger) around the top (bottom) of the layer. Due to the calculated shorter penetration depths for incident wavelengths of 400 nm and 520 nm, incident photons are absorbed around the top of the RPP layer, wherein the weak electric field and the weak vertical carrier transport through the BA barriers lead to low responsivity values. However, for incident wavelength of 450 nm, the longer penetration depth leads to significant photogeneration in the vicinity of the bottom electrodes, wherein the electric field efficiently separates the photogenerated electrons and holes, so that they can be collected by lateral electrodes, leading to the maximum responsivity and detectivity at layer thickness of 800 nm (Fig. 7b). Regarding the absorption spectra for thinner RPP layers, corresponding to precursor molarities lower than 0.5 molar (Fig. 4j), we observe a significant absorption dip around 470 nm (blue range). This behavior is in agreement with the observed severe reduction of blue responsivity of metal/RPP/metal photodetectors based on RPP layer thickness of 300 nm in Fig. 7b, so that blue responsivity has fallen close to the green and UV responsivities, and a nearly uniform broadband photodetection is achieved in the 520–400 nm range. More details on the optoelectronic characteristics of the Al/RPP/Al photodetector with RPP layer thickness of 1500 nm and 300 nm are presented in Figures S3 and S5 in the Supporting Information, respectively.

**Figure 8:**
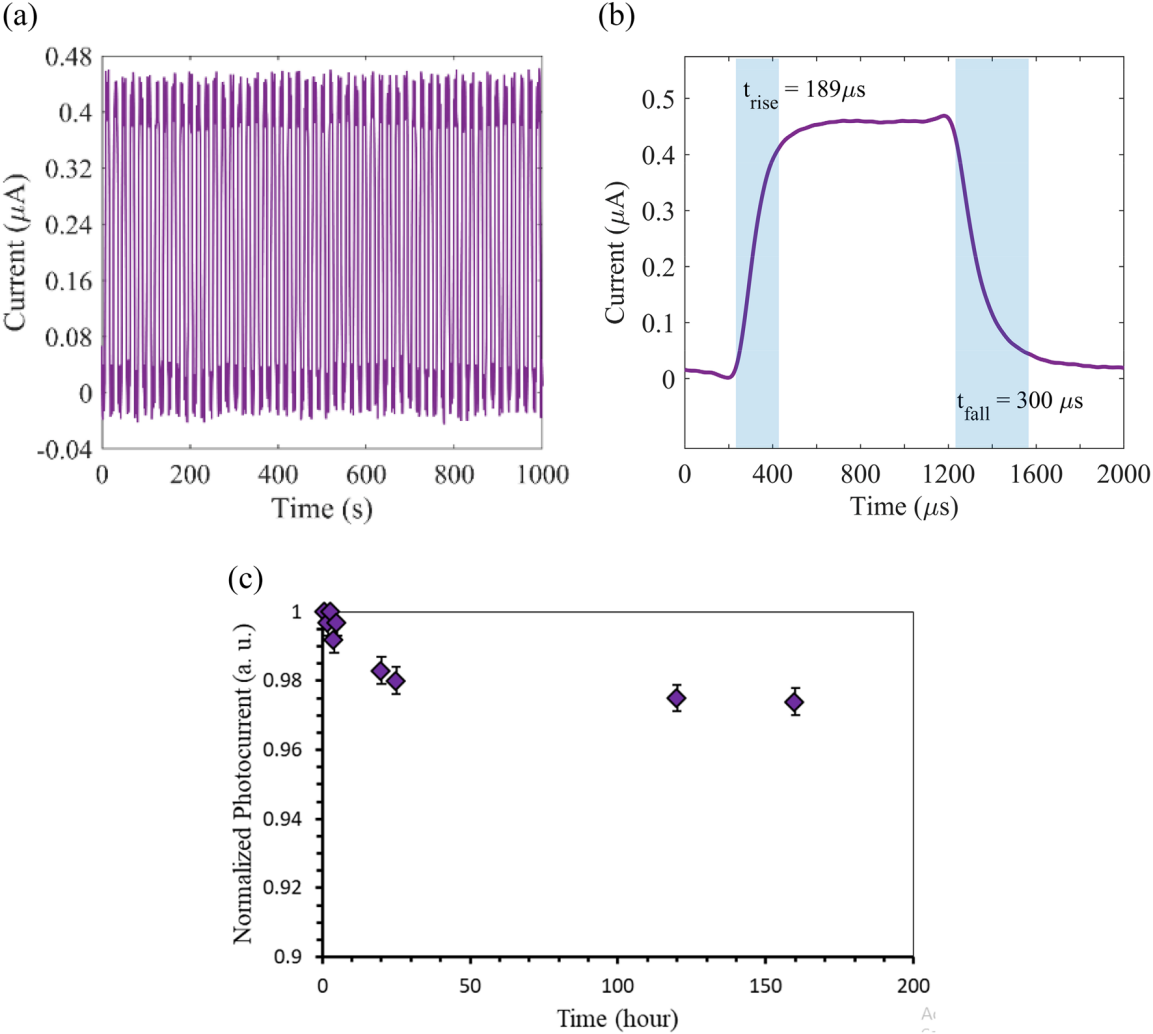
The time resolved photoresponses of the fabricated photodetector, based on RPP layer thickness of 800 nm. (**a**) The measured output photocurrent pulses in response to multiple ON/OFF cycles of UV-illumination, at a fixed power density of 6 mW/cm^2^, and bias voltage of 15 V, revealing a good photodetection stability. (**b**) A time-resolved single photocurrent pulse, revealing a μs-scale fast response. (**c**) Stability of the photodetection behavior, revelaing the normalized photocurrent versus time, over a week.

Moreover, the time resolved photoresponse of the photodetector based on RPP layer with thickness of 800 nm is shown in Fig. 8. Figure 8a shows the measured photoresponse to multiple ON/OFF cycles of light illumination at wavelength of 400 nm and power density of 6 mW/cm^2^, demonstrating a repeatable and stable operation behavior of the realized photodetector. To elaborate the response time of the realized device, we present the time resolved photocurrent in response to a single pulse of UV illumination with the same power density in Fig. 8b. As observed, the rise time and fall times are measured 189 µs, and 300 µs. In order to calculate the rise (fall) time, we measured the time duration required for transition from 10% (90%) to 90% (10%) of the photocurrent. The measured response time values confirm the fast response behavior of our realized Al/RPP/Al photodetector, which is about one order of magnitude faster than the reported photodetectors based on polycrystalline RPP thin films^30,31,55,65–68^. The observed fast response, high responsivity and detectivity are attributed to the described optimization in the hot cast process, leading to a high quality, uniform and close packed crystal grains with dimensions comparable with the metal electrodes spacing in the fabricated device, in addition to the realized back-to-back Al/RPP Schottky junctions in the presented MSM photodetector. It is notable that the response time of our realized hot cast RPP-based photodetector is comparable with the previously reported photodetectors based on bulk single crystal RPPs^33,35,69^. However, our presented fast response RPP-based photodetector takes advantage of a simple, low-cost, and controllable hot cast that allows depositing high quality polycrystalline RPP layers on different platforms, such as glass as a stable and low-cost substrate in the present work.

Moreover, the presented photodetector benefits from high responsivity and detectivity, which can lead to a blue-sensitive or an approximately uniform broadband photodetection, depending mainly on the layer thickness and hot cast parameters. Table 1 displays a summary of photodetection performance of the present work and the other previously reported RPP-based photodetectors. Considering the achieved promising low-cost and photodetection performance in visible range, the present fast response RPP-based photodetectors is proposed as a suitable candidate for applications like visible light communications.

**Table 1:** Performance of photodetectors based on Ruddlesden–Popper perovskite.

RPP material	Configuration	Responsivity (A/W)	Detectivity (Jones)	Rise/fall time (ms)	Wavelength (nm)	Bias (V)	Contact	Ref
(BA)_2_MA_n−1_Pb_n_I_3n+1_/MAPbI_3_	Vertical	0.184	8 × 10^10^		White light		Au	^27^
(PEA)_2_MA_3_Pb_4_I_13_	Vertical	0.46	6 × 10^11^	5.8/4.6	600		Au	^26^
(BA)_2_MA_2_Pb_3_I_10_	Lateral	0.013	NA	10/7.5		30	Au	^30^
(iBA)_2_MA_3_Pb_4_I_13_	Lateral	0.117	NA	16/15	532	1.5	Au	^65^
(BA)_2_MA_n−1_Pb_n_Br_3n+1_	Lateral	0.19	NA	210/240	500	1	Au	^67^
(BA)_2_MAPb_2_I_7_ /(BA)_2_PbI_4_	Lateral	8.12	1.5 × 10^12^	NA	460	30	Au	^34^
(OA)_2_FA_n−1_Pb_n_Br_3n+1_	Lateral	32		0.25/1.45	442	9	Au	^42^
(PEA)_2_MA_n−1_Pb_n_I_3n+1_ single crystal	Lateral	0.25	8.6 × 10^12^	NA	500	5	Au	^70^
(PEA)_2_SnI_4_	Lateral	16	1.9 × 10^11^	630/3600	470	5	Au	^68^
(PEA)_2_PbBr_4_ single crystal	Lateral	98	1.6 × 10^15^	0.06/0.05	460	4	Au	^35^
(PEA)_2_MA_n−1_Pb_n_I_3n+1_/MAPbI_3_	Lateral	149	2 × 10^12^	69/103	598	9	Au	^31^
BA_2_MA_2_Pb_3_I_10_	Lateral	10	NA	NA	528	6	Au	^56^
BA_2_PbBr_4_/graphene	Lateral	2100	NA	NA	470	0.5	Au	^46^
(PEA)_2_SnI_4_/MoS_2_	Lateral	1100	NA	34/38	451	3	Au	^66^
(PEA)_2_PbBr_4_ single crystal	Lateral	0.034	9.03 × 10^10^	0.41/0.37	365	10	Au	^33^
(C_4_H_9_NH_3_)_2_(CH_3_NH_3_)Pb_2_I_7_	Lateral	8.4	1.2 × 10^12^	NA	460	30	Au	^32^
BA_2_PbBr_4_ single crystal	Lateral	0.000381	9.01 × 10^10^	NA	447	5	Au	^54^
(PEA)_2_PbI_4_	Lateral	1.07	2.96 × 10^11^	44/45	460	3	Au/ gr	^55^
(BA)_2_PbBr_4_ single crystal	Lateral	0.0169	2.06 × 10^12^	0.22/0.24	377	10	Ag	^69^
(BA)_2_PbI_4_	Lateral	0.119	2.15 × 10^8^	0.189/0.3	450	15	Al	This work

To elaborate the photodetection stability, we have investigated photocurrent of the realized RPP-based photodetector after different time durations, and ploted the normalized photocurrent versus time, as shown in Fig. 8c. For this purpose, we kept the sample in the ambient environment and dark condition, between the measurements, and applied illumination wavelength of 520 nm, power density of 2 mW/cm^2^, and bias voltage of 10 V at the illumination cycles. It is notable that the investigated sample is not encapsulated, without using any passivation layer on the 2D RPP layer, directly exposed to the environment in the presented MSM photodetector with lateral configuration. Our experiments show that the realized photodetector shows reasonable stability with about 2.5% degradation over multiple exposures to illumination cycles during about a week. It is well established that encapsulating the device can enhace the stability significantly^5^.

## Conclusions

In this paper, we initially studied the effect of different parameters of hot casting BA_2_PbI_4_ layer on the optical and optoelectronic properties, in addition to layer morphology and thickness. We explored the effect of substrate temperature, rotational speed, plasma treatment, and RPP/DMF molarity on formation of the RPP layer. The hotter substrate and higher rotational speed as well as plasma treatment have led to better crystallization of the RPP layer, due to higher solvent evaporation rate. Crystal growth occurs mainly through supersaturation of precursor solution, so that substrate temperature, rotational speed and plasma treatment determine the crystalline quality and morphology of the achieved layer. The optimum hot casting conditions for realizing photodetection were achieved substrate temperature of 100 °C and rotational speed of 5000 rpm, resulting in close packed crystal gains with average dimension of about 30 × 30 μm^2^. The effect of precursor molarity was more dominate on the layer thickness rather than its morphology, so that lower molarity leads to smaller thickness. Fabricating electrode gaps smaller than the average gain size, we achieved rise time of 189 μs for the realized Al/RPP/Al photodetector, which is much faster than the reported poly crystalline counterparts. We observed a maximum responsivity of 119 mA/W to incident wavelength of 450 nm for RPP layer thickness of 800 nm, due to longer penetration depth of blue light in the active layer. Otherwise, decreasing the RPP layer thickness to 300 nm leads to lower blue absorption, and nearly wavelength-independent broadband photoresponse in wavelength range of 400–520 nm. The presented polycrystalline RPP-based photodetector benefits from a simple and low-cost fabrication process on glass substrate, a good stability and responsivity, and a fast photoresponse, comparable with bulk single crystal RPP-based counterparts that entitle them as promising candidates for wide applications such as visible light communications.

## Experimental section

### Fabrication process

To synthesize the RPP layer, 500 mg of PbO powder is dissolved in a mixture of HI solution (57%, 3 ml), and H3PO2 solution (50%, 1 ml), while heating at 180 °C and stirring for about 5 min, which leads to a bright yellow solution product of PbI_2_. Then, 1.5 ml of n-CH_3_(CH_2_)_3_NH_3_I (BAI) is added to the PbI_2_ solution, initially producing a black precipitate, which is subsequently dissolved by continuing heating at 180 °C. Stirring is stopped after 5 min, and the solution is left to cool down to room temperature, during which orange rectangular-shaped crystalline flakes are formed. Then, using vacuum filtering BA_2_PbI_4_ flakes are separated from the solvent, and dissolved in DMF at 70 °C subsequently to achieve an appropriate and homogeneous precursor solution for hot casting step. It is well known that perovskite layers are incompatible with the processes that involve aqueous solutions, such as optical lithography. Thus, to fabricate the proposed metal/RPP/metal photodetector, we utilized the bottom electrode configuration, and first realized the metal interdigital electrodes on glass substrate. For this purpose, we cleaned the glass substrate by sonication in deionized water and 2-propanol, then exposed it to oxygen plasma for 10 min. As the next step, an Al layer with a thickness of 100 nm was deposited on the substrate by physical vapor deposition (PVD), and patterned to 30 pairs of interdigital electrodes with a length of about 600 µm. Spacing between the fingers and the fingers width are equally 20 µm, so that the whole dimensions of the electrodes pattern is about 1800 × 600 µm^2^. Then, to hot cast the RPP layers, the sample is heated, and transferred immediately on the spin coater to spin the prepared RPP/DMF solution for about 40 s.

### Characterization methods

Surface morphologies of all the samples are characterized with optical microscopy and scanning electron microscopy (SEM). X-ray diffraction (XRD) is used to study the crystalline structure of the prepared layers. UV–Vis absorption and photoluminescence (PL) spectra are acquired to study the optical properties, while an excitation wavelength of 380 nm has been used for PL. To measure the response time of the realized photodetector, a 1 MΩ resistor is applied in series with the investigated photodetector, while it is exposed to ON/OFF illuminating cycles.

## Supplementary Information

Supplementary Information.

## Data Availability

The datasets used and/or analysed during the current study available from the corresponding author on reasonable request.
