# Supplementary material for: Pinhole-free 2D Ruddlesden–Popper perovskite layer with close packed large crystalline grains, suitable for optoelectronic applications
Source: Sci Rep. 2023 May 24;13:8374. doi: 10.1038/s41598-023-35546-1 (PMC10209112; doi:10.1038/s41598-023-35546-1)
Supplement: Supplementary file 1 — Supplementary Information. [file 41598_2023_35546_MOESM1_ESM.docx]

**Supporting Information**

Pinhole-free 2D Ruddlesden-Popper Perovskite layer with close packed large crystalline grains, suitable for optoelectronic applications

**Parsa Darman, Amin** **Yaghoobi, Sara Darbari*^1^**

^1^ Nano-Sensors and Detectors Lab., and Nano Plasmo-photonic Research Group, Faculty of Electrical and Computer Engineering, Tarbiat Modares University, Tehran, Iran.

^*^ [s.darbari@modares.ac.ir](mailto:s.darbari@modares.ac.ir)

| 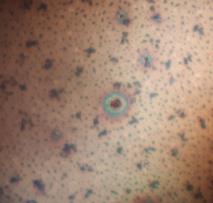 50 µm  (d) 0.018 molar | 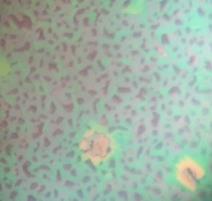 50 µm  (c) 0.026 molar |  50 µm  (b) 0.039 molar |  50 µm  (a) 0.057 molar |
| --- | --- | --- | --- |
| 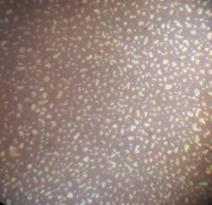 50 µm  (h) 3.9 mmolar |  50 µm  (g) 5.7 mmolar | 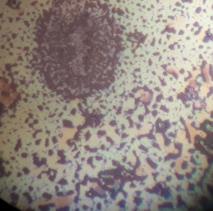 (f) 8.4 mmolar  50 µm |  50 µm  (e) 0.012 molar |
| 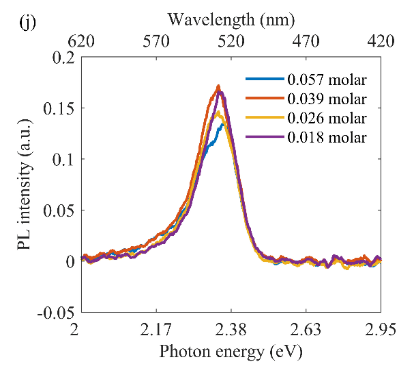 | |  500 nm  (i) 0.057 molar | |
| 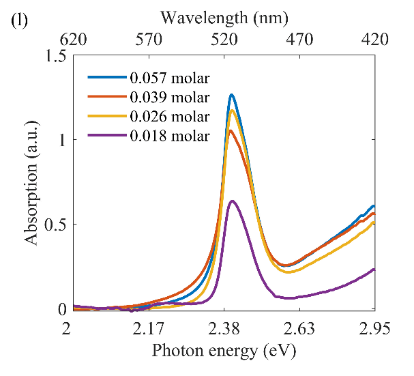 | | 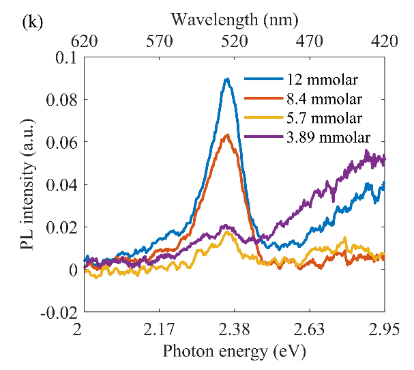 | |

**Figure S1.** (a-h) Optical microscopy images of RPP layers, hot cast by RPP/DMF solution of (a) 0.057 molar, (b) 0.039 molar, (c) 0.026 molar, (d) 0.018 molar, (e) 8.4 mmolar, (f) 5.7 mmolar, (g) 3.89 mmolar, (h) 1.2 mmolar. (i) Cross section SEM image of RPP layer corresponding to 0.057 molar. (j, k) PL spectra of of RPP layers, hot cast by RPP/DMF precursors of (j) 0.057 molar to 0.018 molar, and (k) 12 mmolar to 3.89 mmolar. (l) Absorption spectra of hot cast (BA)_2_PbI_4_ layers with RPP/DMF precursor molarities of 0.057 molar to 0.018 molar.


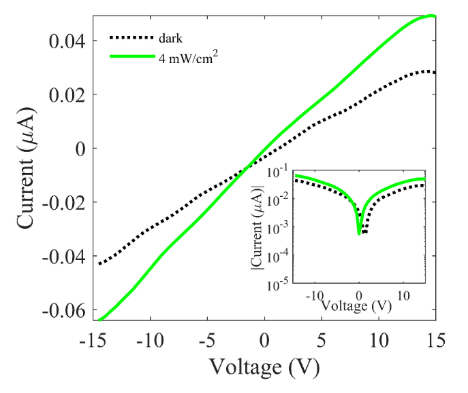


**Figure S2.** The current-voltage characteristics of the fabricated Au/RPP/Au photodetector, in response to power density of 4 mW/cm^2^ and wavelength of 520 nm. The inset shows the current in logarithmic scale.























**Figure S3.** Optoelectrical characteristics of Al/(BA)_2_PbI_4_/Al photodetector with RPP layer thickness of 1500 nm. (a-f) The current voltage characterization in the dark and under illumination of green (520 nm), blue (450 nm), and UV (400 nm) light with optical power of (a) 6 mW/cm^2^ (b) 4 mW/cm^2^ (c) 2 mW/cm^2^ (d) 1.5 mW/cm^2^ (e) 1 mW/cm^2^ (f) 0.5 mW/cm^2^. (g-i) The current voltage characterization of the device under illuminations of (g) UV (400 nm) (h) blue light (450 nm) (i) green (520 nm) light with optical power in range of 0.5 mW/cm^2^ and 6 mW/cm^2^. (j) The photocurrent of (g-i) for every power of light source at 15 V bias under green (520 nm), blue (450 nm), and UV (400 nm) illuminations.



















**



**

**Figure S4.** Optoelectrical characteristics of Al/(BA)_2_PbI_4_/Al photodetector with RPP layer thickness of 800 nm. (a-f) The current voltage characterization in the dark and under illumination of green (520 nm), blue (450 nm), and UV (400 nm) light with optical power of (a) 6 mW/cm^2^ (b) 4 mW/cm^2^ (c) 2 mW/cm^2^ (d) 1.5 mW/cm^2^ (e) 1 mW/cm^2^ (f) 0.5 mW/cm^2^. (g-i) The current voltage characterization of the device under illuminations of (g) UV (400 nm) (h) blue light (450 nm) (i) green (520 nm) light with optical power in range of 0.5 mW/cm^2^ and 6 mW/cm^2^. (j) The photocurrent of (g-i) for every power of light source at 15 V bias under green (520 nm), blue (450 nm), and UV (400 nm) illuminations.























**Figure S5.** Optoelectrical characteristics of Al/(BA)_2_PbI_4_/Al photodetector with RPP layer thickness of 300 nm. (a-f) The current voltage characterization in the dark and under illumination of green (520 nm), blue (450 nm), and UV (400 nm) light with optical power of (a) 6 mW/cm^2^ (b) 4 mW/cm^2^ (c) 2 mW/cm^2^ (d) 1.5 mW/cm^2^ (e) 1 mW/cm^2^ (f) 0.5 mW/cm^2^. (g-i) The current voltage characterization of the device under illuminations of (g) UV (400 nm) (h) blue light (450 nm) (i) green (520 nm) light with optical power in range of 0.5 mW/cm^2^ and 6 mW/cm^2^. (j) The photocurrent of (g-i) for every power of light source at 15 V bias under green (520 nm), blue (450 nm), and UV (400 nm) illuminations.

**Table S1**. X-ray diffraction data of the (BA)_2_PbI_4_ perovskites.

| Position (2θ) | Counts | FWHM (2θ) | d-spacing (A) |
| --- | --- | --- | --- |
| 7.52 | 164587 | 0.1226 | 13.62 |
| 14.96 | 11526 | 0.1308 | 6.86 |
| 22.47 | 18068 | 0.1408 | 4.58 |
| 30.09 | 32567 | 0.1508 | 3.44 |
| 37.84 | 10902 | 0.1639 | 2.75 |
